# The interaction between third molars and surrounding periapical tissues in mandibular stress distribution during high-impact trauma: a finite element study

**DOI:** 10.4317/medoral.26954

**Published:** 2025-01-26

**Authors:** Carlos Bruno Pinheiro Nogueira, Fábio Wildson Gurgel Costa, Francisco Samuel Rodrigues Carvalho, Tácio Pinheiro Bezerra, Ivo Cavalcante Pita Neto, Francisco Ilson da Silva Júnior, Eduardo Costa Studart Soares

**Affiliations:** 1 Postgraduate Student, Postgraduate Program in Dentistry, Federal University of Ceará, Fortaleza, Ceará, Brazil; 2 Professor, Department of Oral and Maxillofacial Surgery, Postgraduate Program in Dentistry, Federal University of Ceará, Fortaleza, Ceará, Brazil; 3 Professor, Department of Oral and Maxillofacial Surgery, Postgraduate Program in Dentistry, UNICHRISTUS, Ceará, Brazil; 4 Professor, Department of Oral and Maxillofacial Surgery, Postgraduate Program in Dentistry, UNILEÃO, Ceará, Brazil; 5 Professor, Department of Mechanical Engineering, Federal University of Ceará, Fortaleza, Ceará, Brazil; 6 Full Professor, Department of Oral and Maxillofacial Surgery, Postgraduate Program in Dentistry, Federal University of Ceará, Fortaleza, Ceará, Brazil

## Abstract

**Background:**

The presence of mandibular third molars has been associated with the risk of mandibular fractures, highlighting the need for comprehensive studies considering the interaction with other mandibular structures. This study investigates how mandibular third molars and neighboring tissues can influence the structural fragility of the mandible using finite element analysis.

**Material and Methods:**

A finite element analysis study following the guidelines proposed by RIFEM 1.0 was performed using three previously created mandible models: Model A, without right and left third molars; Model B, without one third molar; Model C, with bilateral presence of third molars. A 2452N force was applied to the right mandibular body in a virtual environment, allowing for a structural analysis of each mandible.

**Results:**

Models without third molars and with only one third molar showed similar energy dissipation patterns, contrasting with the model with both third molars. The presence of third molars influenced the magnitude and distribution of stress, highlighting fragility points in specific areas such as the lingual surface, the condyles bilaterally (models without and with one contralateral third molar to trauma), and the distal cervical region of the second molar (third molar absent), as well as significantly showed the path of energy towards the contralateral side of the trauma with a concentration of energy at the contact points of virtually all teeth present immediately after impact.

**Conclusions:**

The presence of mandibular third molars influenced the distribution and magnitude of stress within the mandible during a simulated high-impact trauma. Models with third molars exhibit distinct stress patterns, with fragility points appearing in critical areas such as the lingual surface, condyles, and second molar regions. These findings suggest that the presence of third molars increases the structural fragility of the mandible, potentially elevating the risk of mandibular fractures, especially in the context of traumatic impacts.

** Key words:**Computer-assisted image processing, finite element analysis, dental stress analysis, biomechanics, mandible.

## Introduction

The mandible is a vital part of the facial skeleton, essential for masticatory function, aesthetics, and facial structural integrity (1). Its angle, significant for biomechanical function, is prone to trauma that can weaken its strength and stability (1-3). Notably, the presence of mandibular third molars is a factor of considerable clinical and scientific interest concerning mandibular integrity (4).

In dentistry, the relationship between third molars and the fragility of the mandibular angle has sparked considerable debate (4). Studies indicate that these teeth may increase the risk of fractures, prompting essential discussions about prophylactic interventions, such as the preventive removal of third molars, to reduce risks in mandibular trauma scenarios (5-10).

Current literature contains various studies on the relationship between mandibular third molars and the fragility of the mandibular angle (5-10). However, research is lacking that thoroughly examines this fragility by considering not only the presence of these teeth but also their interaction with other mandibular elements, such as the external oblique line, mylohyoid line, retromolar region, and additional structures of the mandibular angle.

Advancements in computational techniques have enhanced the biomechanical analysis of the mandible (11). The Finite Element Method (FEM) serves as a powerful tool for simulating complex phenomena, enabling accurate mathematical modeling of anatomical structures, including maxillofacial bones, joints, and teeth (12), as well as evaluating responses to external stimuli such as force, pressure, temperature variation, and magnetic fields (13).

This study aims to elucidate the impact of mandibular third molars on stress concentration in the mandibular angle, considering the complex structures and tissues involved in bodily trauma through three-dimensional finite element analysis. This approach will enhance understanding of mandibular behavior following trauma, addressing existing gaps in the literature and advancing knowledge of the underlying biomechanics. Ultimately, this study aims to refine clinical practices and guide therapeutic decisions based on solid scientific evidence.

## Material and Methods

This study was conducted with approval from the Local Ethics Committee. It builds upon the work of Bezerra *et al*. (7), systematically dividing the research description into subdomains as recommended by the RIFEM 1.0 guidelines (13). This structured approach aims to enhance clarity and comprehension of the research.

- Finite Element Models

Three distinct mandibular models were prepared for finite element (FE) analysis. The pixels corresponding to the third molars were replaced with spongy and cortical bone, respectively, creating the following models:

Model A: Mandible without the two lower third molars.

Model B: Mandible with the left third molar absent.

Model C: Mandible with both lower third molars intact. All other mandibular structures remained unchanged across the models.

The 3D reconstruction of the mandibles was carried out using Scan IP® (Simpleware Ltd, Exeter, UK), which converted the models into mesh-based simulations. These meshes were subsequently exported to ANSYS® (SIMULIA, Providence, RI, USA, version 13.0) for structural analysis of the mechanical tests.

- Mesh Generation

The final models were patient-specific and highly detailed. A robust mesh of interconnected triangular and tetrahedral finite elements was created to simulate the mechanical behavior of the mandible, facilitating an integrated analysis of the various structural interactions within the mandibular complex. The volume and density of the finite elements were consistent with those in Bezerra *et al*. (7).

- Development of the Finite Element Model (FEM)

a. Image Acquisition

The models were constructed using cone-beam computed tomography (CBCT) scans (Veraview X800, J. MORITA MFG. CORP., Japan) from a 30-year-old male patient with no history of orthodontic treatment, mandibular pathology, or fractures, and with all lower teeth in excellent condition, as detailed by Bezerra *et al*. (7).

b. Dimensional Type of FEM

A 3D model was generated after importing the DICOM (Digital Imaging and Communications in Medicine) data. This allowed for the evaluation of multiple variables across structures and load conditions in different directions.

c. Software and Segmentation Process

The Scan IP (Simpleware Ltd) software was used to generate the mesh via a discretization process. Multiple masks were created and segmented based on the CBCT data, representing various anatomical structures, including enamel, dentin, pulp, the periodontal ligament, cortical bone, and spongy bone. Soft tissues were not included in this analysis.

d. Mesh Elements and Properties

Each discretized mask adhered to isotropic mechanical properties, assuming homogeneous and linear-elastic deformation patterns. The Young's modulus and Poisson's ratio for each mask followed the previous study (7).

e. Boundary Conditions and Interface Constraints

Boundary conditions were established by immobilizing the most posterior and superior points of the condyles in all directions. Masticatory muscle actions were simulated using fan-shaped resistance elements with vector forces, as outlined by Bujtár *et al*. (14). This approach incorporated muscle stiffness based on estimated tissue deformation (Supplement 1).

f. Load Application and Trauma Simulation

**Figure 1:**
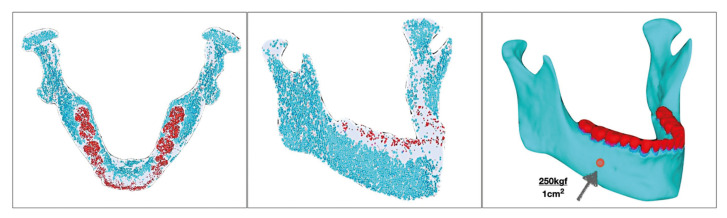
A blunt trauma, perpendicularly to the mandibular body with 250kgf /1cm2 circular.

A blunt-force trauma simulation was performed on the right mandibular body, applying a load of 250 kgf (2452 N) over a 1 cm² area. This force mimicked real-life traumatic impact scenarios, such as assaults or collisions, as noted by Bezerra *et al*. (8). The force was applied 1 cm from the most external and posterior point of the right mental foramen to evaluate the static behavior of the mandible under stress (Fig. 1).

## Results

**Figure 2:**
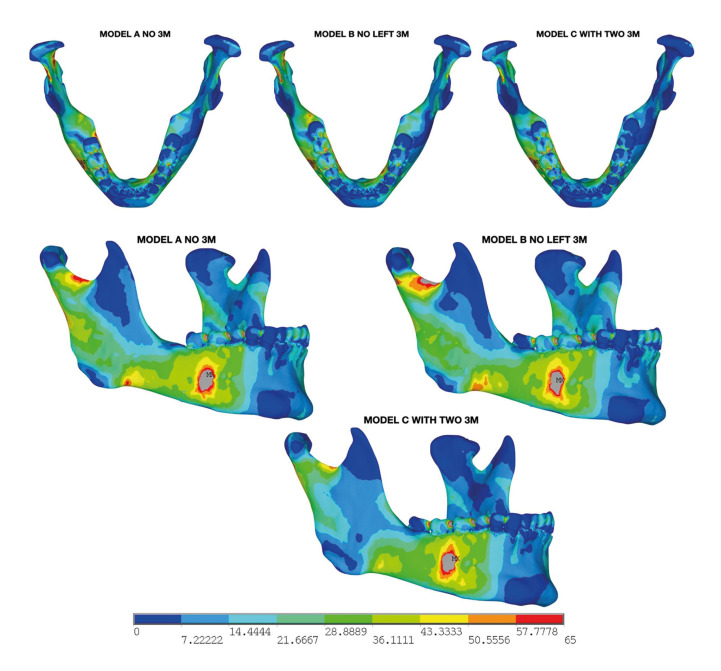
Overall comparative result between models using a colorimetric scale.

The patterns of energy dissipation after trauma showed distinct variations among the analyzed models, highlighting both unique and overlapping mandibular movement characteristics. This variation was assessed using von Mises stress distributions (in MPa), illustrated in a colorimetric map for clear visualization of stress zones. We evaluated energy transfer dynamics by examining each mandible model from the trauma epicenter, tracking changes from peak stress (gray) to minimum stress (blue). Notably, energy displacement was more similar between models without third molars (Model A) and those with one third molar (Model B) than in the model with bilateral third molars (Model C) (Fig. 2).

The presence of third molars (3M) significantly affected the magnitude and distribution of energy after trauma. This influence extended beyond the third molars, as surrounding teeth and tissues and their contact points were vital in dissipating forces. The observed interactions indicate that the mandibular response to blunt trauma involves a broader biomechanical network rather than being localized.

**Figure 3:**
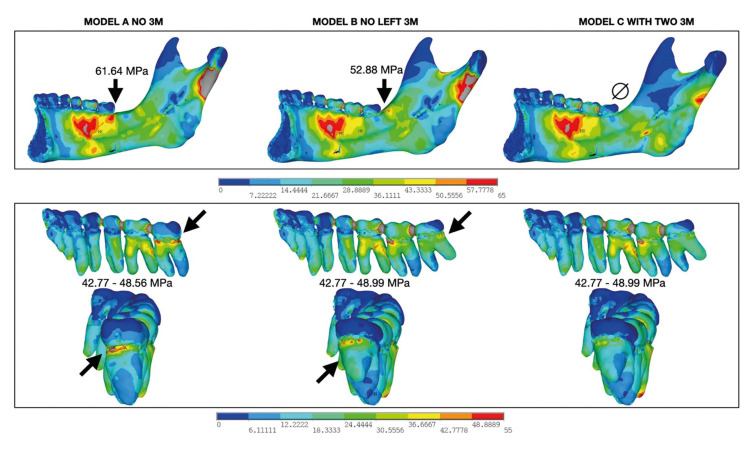
Relationship between energy concentration and points of possible fragility in the mandibular angle.

A key difference in stress amplitude was noted in the mandibular angle region across the models. In the model without third molars, the von Mises stress in the distal lingual area of the second molar (2M) was approximately 61.64 MPa, with values ranging from 42.77 to 48.56 MPa in the distal cervical region. In the model with one third molar, similar stress patterns emerged, but with three distinct stress concentration regions. Von Mises values near the third molar ranged from 42.77 to 48.99 MPa, with 52.88 MPa distal to the right third molar. However, in the model with bilateral third molars, no significant stress was observed in these regions after the simulation (Fig. 3).

Fragility points were consistently identified at the impact site and along the lingual surface near the trauma region, as well as in both ipsilateral and contralateral condyles. In all models, these areas exhibited von Mises stress values above 65 MPa, indicating a high susceptibility to structural failure. Additionally, energy displacement patterns showed pronounced flow along the mandible's lingual surface, highlighting the relationship between mandibular morphology and impact absorption capacity (Supplement 2).

**Figure 4:**
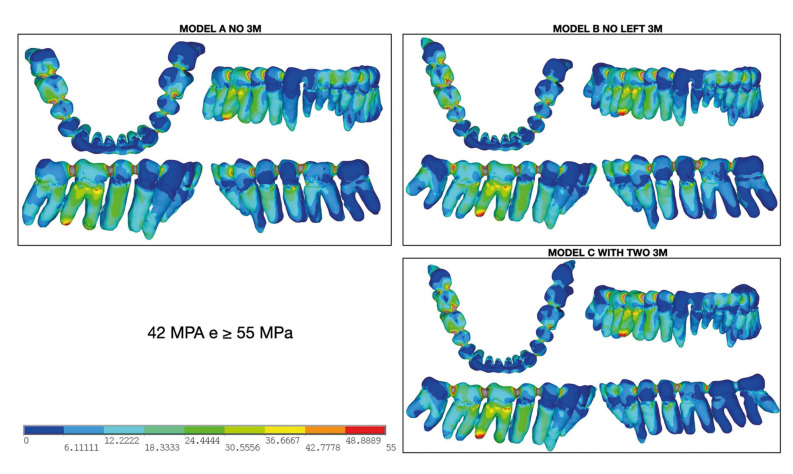
Distribution of energy among the teeth through contact points along the mandible.

Another noTable finding was the uniform energy distribution across dental contact points from the trauma epicenter to the contralateral side. Stress values in these areas ranged from 42 MPa to over 55 MPa, leading to stress concentrations at nearly every tooth contact point. Furthermore, the root region of the directly impacted tooth exhibited considerable fragility in all models. These findings underscore the critical role of teeth in stress concentration and distribution, as well as their potential contribution to fracture propagation during mandibular trauma (Fig. 4).

## Discussion

The results of this study are based on the observation and comparison of a computational simulation that employed finite element analysis (FEA) on a patient-specific, high-resolution custom mandibular model. This model included dental tissues (cementum, dentin, enamel, pulp, and periodontal ligament) to assess the distribution of energy under tension and compression following trauma to the body of the mandible, as well as the dynamic relationship between this trauma and the stress distribution in the bone.

The findings indicate that third molars (3Ms) significantly affect energy dissipation after mandibular trauma. Models lacking 3Ms or with only one exhibited similar energy patterns, while models with both 3Ms showed distinct behaviors. This suggests that 3M quantity and position influence the mandible's biomechanical response to trauma. Vulnerability points were identified on the ipsilateral lingual surface near impact sites and in the condyles, marking critical fracture locations during mandibular trauma. These results highlight the intricate relationship among mandibular morphology, energy distribution, and the presence of teeth, offering insights into preventing mandibular fractures in blunt trauma.

Contrarily, observational studies report a higher risk of angular fractures with 3Ms present and condylar fractures when absent (11,15-17). Although conflicting evidence exists regarding dental impaction's role in fracture occurrence, some studies suggest fully erupted 3Ms may correlate with increased condylar fractures (18,19). Notably, Mohammed Al-Sharani *et al*. (20) found that a lack of occlusal support heightened the risk of condylar fractures, independent of 3M characteristics (20). Further research is needed to clarify the interplay between fully erupted 3Ms and condylar/angular fractures.

Computational analyses using finite element techniques have explored these complex relationships (7). Antic *et al*. (8) noted that during lateral trauma, both the mandibular angle and ipsilateral condyle are more prone to fractures, with the ipsilateral condyle being especially vulnerable, regardless of 3M presence. In frontal impacts, angular regions show increased fracture susceptibility with fully erupted 3Ms, which intensifies with partial impaction. Without 3Ms, condyles become the most fracture-prone areas (8).

The present findings enhance the understanding of the biomechanics of mandibular trauma and the role of 3Ms. Both mandibular angle and ipsilateral condyle are vulnerable to fractures during lateral trauma, while frontal trauma reveals angular regions more susceptible in the presence of fully erupted 3Ms (8). Bezerra *et al*. (7) corroborate that with one 3M, stress shifts to the contralateral condyle, while its absence heightens condylar fragility. Thus, the presence and position of 3Ms, along with mandible morphology and impact area, critically influence stress distribution and fracture risk.

Further studies have examined lateral force impacts through finite element analysis, confirming stress concentration in the mandibular angle (5,9,10). Takada *et al*. (5) reported that lateral impacts on mandibles with partially erupted 3Ms cause stress to center around the tooth root apex, affecting the mandibular angle. Kılınç *et al*. (9) found that vertical impaction increases angle fragility during trauma to the mandible's body. They proposed that fully erupted or impacted lower 3Ms are risk factors for angle fractures but protect the condyle (9), whereas their absence strengthens the angle and poses a risk to the condyle (21,22).

Despite our findings showing higher stress in the condylar region even with 3Ms present, our study was limited to fully erupted 3Ms and lateral force impacts in the mandibular body. Future research should evaluate the influence of varying angles and degrees of impaction. This study offers valuable insights into stress distribution across the mandibular arch, pinpointing vulnerabilities at contact points and root areas.

These findings may impact treatment decisions following facial trauma. Dental professionals should conduct thorough radiographic evaluations, particularly using cone-beam computed tomography, to identify hidden root fractures in impacted areas (23,24). Discussions around managing teeth within fracture lines suggest that retaining intact teeth in these spaces rarely leads to complications (25,26). However, tooth prognosis relates to dental mobility, periodontal health, and bone displacement (27). Therefore, unless clear evidence of a fracture or structural alteration is present, a conservative management approach may be indicated.

## Conclusions

The presence of mandibular third molars influenced the distribution and magnitude of stress within the mandible during a simulated high-impact trauma. Models with third molars exhibit distinct stress patterns, with fragility points appearing in critical areas such as the lingual surface, condyles, and second molar regions. These findings suggest that the presence of third molars increases the structural fragility of the mandible, potentially elevating the risk of mandibular fractures, especially in the context of traumatic impacts.

## Data Availability

The datasets used and/or analyzed during the current study are available from the Correspondence.

## References

[1] Vollmer D, Meyer U, Joos U, Vègh A, Piffko J (2000). Experimental and finite element study of a human mandible. J Craniomaxillofac Surg.

[2] Inaoka SD, Carneiro SC, Vasconcelos BC, Leal J, Porto GG (2009). Relationship between mandibular fracture and impacted lower third molar. Med Oral Patol Oral Cir Bucal.

[3] Ma'aita J, Alwrikat A (2000). Is the mandibular third molar a risk factor for mandibular angle fracture?. Oral Surg Oral Med Oral Pathol Oral Radiol Endod.

[4] Meisami T, Sojat A, Sàndor GK, Lawrence HP, Clokie CM (2002). Impacted third molars and risk of angle fracture. Int J Oral Maxillofac Surg.

[5] Takada H, Abe S, Tamatsu Y, Mitarashi S, Saka H, Ide Y (2006). Three-dimensional bone microstructures of the mandibular angle using micro-CT and finite element analysis: relationship between partially impacted mandibular third molars and angle fractures. Dent Traumatol.

[6] Bezerra TP, Studart-Soares EC, Pita-Neto IC, Costa FW, Batista SH (2011). Do third molars weaken the mandibular angle?. Med Oral Patol Oral Cir Bucal.

[7] Bezerra TP, Silva FI, Scarparo HC, Costa FWG, Studart-Soares EC (2013). Do erupted third molars weaken the mandibular angle after trauma to the chin region?. A 3D finite element study. Int J Oral Maxillofac Surg.

[8] Antic S, Vukicevic AM, Milasinovic M, Saveljic I, Jovicic G, Filipovic N (2015). Impact of the lower third molar presence and position on the fragility of mandibular angle and condyle: A Three-dimensional finite element study. J Craniomaxillofac Surg.

[9] Kılınç Y, Zor ZF, Tümer MK, Erkmen E, Kurt A (2018). Does the angulation of the mandibular third molar influence the fragility of the mandibular angle after trauma to the mandibular body? A three-dimensional finite-element study. Comput Methods Biomech Biomed Engin.

[10] Seyrek NK, Kahraman OE (2022). The effect of different positions of unerupted lower third molar teeth on the fragility of mandibular angle: Finite element analysis. Niger J Clin Pract.

[11] Lee Y, Kim J, Lee M, Shin D, Choi H (2019). Relationship between mandible fractures and third molars. Arch Craniofac Surg.

[12] Wong RC, Tideman H, Merkx MA, Jansen J, Goh SM, Liao K (2011). Review of biomechanical models used in studying the biomechanics of reconstructed mandibles. Int J Oral Maxillofac Surg.

[13] Mathur VP, Atif M, Duggal I, Tewari N, Duggal R, Chawla A (2022). Reporting guidelines for in-silico studies using finite element analysis in medicine (RIFEM). Comput Methods Programs Biomed.

[14] Bujtár P, Sándor GK, Bojtos A, Szucs A, Barabás J (2010). Finite element analysis of the human mandible at 3 different stages of life. Oral Surg Oral Med Oral Pathol Oral Radiol Endod.

[15] Choi BJ, Park S, Lee DW, Ohe JY, Kwon YD (2011). Effect of lower third molars on the incidence of mandibular angle and condylar fractures. J Craniofac Surg.

[16] Brucoli M, Romeo I, Pezzana A, Boffano P, Benech A (2020). The relationship between the status and position of third molars and the presence of mandibular angle and condylar fractures. Oral Maxillofac Surg.

[17] Kandel L, Mishra R, Yadav D, Tripathi S, Shubham S, Chhetri P (2021). Impact of mandibular third molars on angle fractures: A retrospective study. Dent Traumatol.

[18] Soós B, Janovics K, Tóth Á, Di Nardo MD, Szalma J (2020). Association between third molar impaction status and angle or condylar fractures of the mandible: A retrospective analysis. J Oral Maxillofac Surg.

[19] Venkatachalam V, Pandiarajan R (2022). Does the Impacted Mandibular Third Molar Increase the Risk of Angle Fracture to Prevent the Incidence of Condylar Fracture? - A Retrospective Analysis. Ann Maxillofac Surg.

[20] Mohammed Al-Sharani H, Bin Z, Ahmed Mashrah M, Galvão EL, Ahmed Al-Moraissi E, Ali Al-Aroomi M (2021). The influence of wisdom tooth impaction and occlusal support on mandibular angle and condyle fractures. Sci Rep.

[21] Nogami S, Yamauchi K, Bottini GB, Kouketsu A, Otake Y, Sai Y (2018). Do mandibular third molars play a role in fractures of the mandibular angle and condyle?. J Craniofac Surg.

[22] Semel G, Emodi O, Ohayon C, Ginini JG, Rachmiel A (2020). The influence of mandibular gonial angle on fracture site. J Oral Maxillofac Surg.

[23] İlgüy D, İlgüy M, Fişekçioğlu E, Bayirli G (2009). Detection of jaw and root fractures using cone beam computed tomography: a case report. Dentomaxillofac Radiol.

[24] Dölekoğlu S, Fişekçioğlu E, İlgüy D, İlgüy M, Bayirli G (2010). Diagnosis of jaw and dentoalveolar fractures in a traumatized patient with cone beam computed tomography. Dent Traumatol.

[25] Ulbrich N, Ettl T, Waiss W, Gosau M, Moralis A, Reichert TE (2016). The influence of third molars in the line of mandibular angle fractures on wound and bone healing. Clin Oral Investig.

[26] Walker LJ, Koba S, Demiroglu A, Saulacic N, Burkhard JP (2023). Retention of teeth in the fracture gaps of the mandible: a retrospective analysis. Clin Oral Investig.

[27] Lee W, Kim Y, Shin S, Lee J (2021). Prognosis of teeth in mandibular fracture lines. Dent Traumatol.

